# A Systematic Optimization Method for Permanent Magnet Synchronous Motors Based on SMS-EMOA

**DOI:** 10.3390/s24092956

**Published:** 2024-05-06

**Authors:** Bo Yuan, Ping Chen, Ershen Wang, Jianrui Yu, Jian Wang

**Affiliations:** Civil Aviation College, Shenyang Aerospace University, Shenyang 110136, China; 18204282915@163.com (B.Y.); wes2016@sau.edu.cn (E.W.); 13682924808@163.com (J.Y.); w145730@163.com (J.W.)

**Keywords:** permanent magnet synchronous motors, heuristic optimization, structure design

## Abstract

The efficient design of Permanent Magnet Synchronous Motors (PMSMs) is crucial for their operational performance. A key design parameter, cogging torque, is significantly influenced by various structural parameters of the motor, complicating the optimization of motor structures. This paper proposes an optimization method for PMSM structures based on heuristic optimization algorithms, named the Permanent Magnet Synchronous Motor Self-Optimization Lift Algorithm (PMSM-SLA). Initially, a dataset capturing the efficiency of motors under various structural parameter scenarios is created using finite element simulation methods. Building on this dataset, a batch optimization solution aimed at PMSM structure optimization was introduced to identify the set of structural parameters that maximize motor efficiency. The approach presented in this study enhances the efficiency of optimizing PMSM structures, overcoming the limitations of traditional trial-and-error methods and supporting the industrial application of PMSM structural design.

## 1. Introduction

Permanent Magnet Synchronous Motors (PMSMs) have long been integral to advancements in electronics and electrical engineering, distinguished by their high power density performance—a primary focus of both application and research [1,2,3]. The quest for optimal application efficiency in PMSMs hinges on the strategic structural design, wherein cogging torque emerges as a pivotal factor. Variability in the air gap permeability of the stator slot, leading to cogging torque, induces motor vibration and noise, detrimentally affecting control precision. Consequently, the mitigation of cogging torque has garnered focused research efforts, aiming to refine the structural parameters of PMSMs to suppress this undesired effect [4,5,6].

The selection of these parameters, however, is beleaguered by their complexity and abundance, coupled with their interrelated and nonlinear nature. Historically, the determination of structural parameters for PMSMs has relied heavily on finite element simulations or manual calculations—a practice fraught with inefficiencies. With the burgeoning interest in electric aircraft, the development of high power density PMSMs, necessitating reduced cogging torque, has become critically important.

Addressing this need, various studies have explored methodologies to minimize cogging torque through strategic parameter optimization. Reference [7] leverages the energy method and Fourier series analysis, based on the air gap permeability and magnetic flux density distribution of an equivalent slotless motor, to derive a generalized analytical expression for cogging torque. This analytical approach facilitated the identification of optimal design parameters, such as slot–pole combinations and skew angles, subsequently validated via finite element analysis. Similarly, reference [6] examines a four-pole 24-slots surface-mounted PMSM (SPMSM), employing the energy method to analyze cogging torque generation mechanisms and determine optimization parameters. The ensuing simulation and experimental validation underscored notable improvements in motor efficiency, cogging torque reduction, and torque ripple minimization. Furthermore, the influence of magnetic pole edge effects on cogging torque has been scrutinized, and innovative methods have been proposed to modulate the amplitude and phase shift of cogging torque generated by each pole segment [8]. This approach, which aims to minimize cogging torque in stepped-tilted structures, reinforces the importance of precision in the motor design process. Additionally, the refinement of cogging torque calculation methods to enhance accuracy reflects the ongoing evolution of PMSM optimization strategies [9].

Although researchers have done a lot of work in the optimization of motor structure parameters, these methods are almost all from the perspective of trial and error, and the optimization cost is high and the efficiency low. The heuristic algorithm for multi-objective optimization provides an effective solution to this kind of problem. From this point, our work involves a genetic algorithm-based approach for structure optimization, augmenting traditional methodologies with intelligent, data-driven strategies. Nevertheless, the conventional genetic algorithms’ efficiency remains a challenge, prompting the adoption of the SMS-EMOA algorithm to enhance optimization processes [10]. This novel strategy prioritizes the maximization of dominated super volume, employing a selection operator rooted in non-dominated sorting to yield a well-distributed set of solutions across the Pareto front.

The comparative analysis of SMS-EMOA with bleeding-edge methodologies across various benchmarks, including aerospace applications, underscores its efficacy in handling multi-objective optimization challenges [11]. This paper delves deeper, proposing a refined SMS-EMOA model to expedite and elevate the quality of optimization, particularly in reducing cogging torque. It posits a dual-phase optimization strategy—the initial optimization of stator–rotor structural parameters followed by a focused optimization of significant parameters through response surface methodology, culminating in the application of SMS-EMOA to ascertain optimal parameter values.

Addressing the current optimization challenges, namely the extensive parameterization of motor structures and the absence of a rapid motor simulation calculation model, alongside the high coupling of motor structure parameters, this paper introduces a novel motor simulation genetic algorithm optimization model. There are two innovative points in this paper:

(1) An efficiency simulation method of PMSM based on finite element is established, and the data set of permanent magnet motor structure optimization is formed. It can provide support for motor structure optimization.
(2) An intelligent optimization model for motor structure parameters based on the SMS-EMOA method is established, which is named the Permanent Magnet Synchronous Motor Self-Optimization Lift Algorithm (PMSM-SLA).

This paper is organized as follows: fundamental equations of cogging torque and an optimization analysis of the SMS-EMOA are given in the next section. After that, a new PMSM case is studied in Section 3 for method validation, and the optimization method is compared with conventional FEA-based optimization. Finally, Section 4 concludes the paper.

## 2. Methodology

Optimizing the structure of motor slots can enhance motor performance, efficiency, and output power [12]. However, the parameters influencing motor structure are numerous and intricate. In response, this paper establishes a systematic approach for motor structure optimization, grounded in an analysis of the physical characteristics that affect motor efficiency, from the perspective of the motor’s physical properties. This methodology is divided into two steps: (1) analyzing the fundamental physical characteristics of the motor structure and defining decision variables and goals for motor efficiency optimization, and (2) optimizing motor structure parameters using an enhanced heuristic optimization algorithm.

### 2.1. Physical Analysis of PMSM

**Figure 1:**
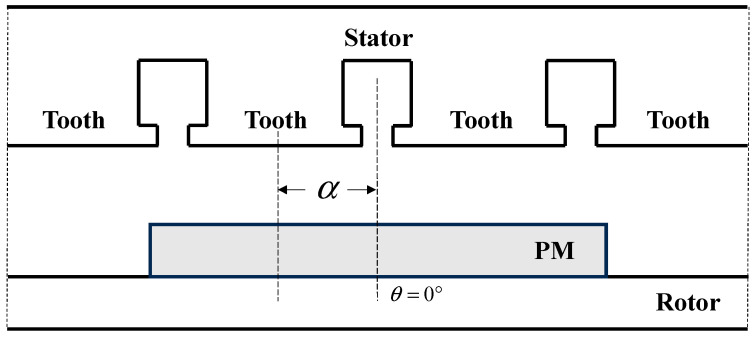
Analysis model of the surface-mounted PMSM(SPMSM).

The relative position of the stator and rotor of the SPMSM is shown in Figure 1. The position of $\theta =0^{{}^{\circ}}$ is defined as the midline of the specified PM. The angle between the center line of a stator tooth and the center line of the specified PM is designated as α. Neglecting the magnetic potential drop on the stator core, the air-gap flux density distribution along the circumference can be expressed as Equation (1):

(1) $$B_{ag}\left(\theta ,\alpha \right)=\mu_{0}\frac{F\left(\theta \right)}{h_{c}+\delta \left(\theta ,\alpha \right)}$$

where F(θ), $h_{c}$, and δ(θ,α) represent the distribution of PM thickness, the air-gap magnetomotive force (AMF), and the effective air-gap length along the circumference, respectively. The magnetic co-energy stored in the air gap [9] can be described as Equation (2):

(2) $$W_{ag}=\frac{1}{2\mu_{0}}\int_{V}{\left[B_{ag}\left(\theta ,\alpha \right)\right]}^{2}dV=\frac{\mu_{0}}{2}\int_{V}F^{2}\left(\theta \right){\left[\frac{1}{h_{c}+\delta \left(\theta ,\alpha \right)}\right]}^{2}dV$$

Therefore, the cogging torque can be calculated by Equation (3):

(3) $$T_{cog}=-\frac{\partial W_{ag}}{\partial \alpha }$$

The Fourier expansion of $F^{2}\left(\theta \right)$ on the interval $\left[-\frac{\pi }{2p},\frac{\pi }{2p}\right]$ can be expressed as Equation (4):

(4) $$F^{2}\left(\theta \right)=F_{0}+\sum_{n=1}^{\infty }F_{n}\cos\left(2np\theta \right)$$

where $F_{0}=\alpha_{p}F^{2}$, $F_{n}=\frac{2}{n\pi }F^{2}\sin\left(n\alpha_{p}\pi \right)$, $F=\frac{B_{r}h_{c}}{\mu_{0}}$. $\alpha_{p}$ and $B_{r}$ are the pole–arc coefficient and the remanent magnetization of the PM, respectively. Taking into account the influence of the stator slot structure on the air-gap permeance, the effective air-gap length can be calculated using the magnetostatic solver of finite element software. The magnetic potential within a tooth pitch is practically identical to that at the tooth centerline due to the inner surface of the stator within a tooth pitch being an equal magnetic potential surface. The suitable vector magnetic potential is given at the left and right boundaries of the calculation model for the effective air-gap length, as demonstrated in [9]. The magnetic potential within a tooth pitch is basically the same as the magnetic potential $F_{\delta t}$, which can be obtained using Equation (5):

(5) $$F_{\delta t}=\frac{B_{\delta tN}\delta^{\prime }}{\mu_{0}}$$

where $B_{\delta tN}$ is the flux density at point N and $\delta^{\prime }$ is the air-gap length at the tooth centerline. The effective air-gap length within a tooth pitch can be obtained with Equation (6):

(6) $$\delta \left(\theta ,\alpha \right)=\frac{\mu_{0}F_{\delta t}}{B_{\delta t}\left(\theta ,\alpha \right)}-h_{c}$$

The distribution of the air-gap flux density within a tooth pitch is represented as $B_{\delta t}\left(\theta ,\alpha \right)$. This method can be employed to efficiently and precisely determine the effective air-gap length and air-gap permeance in electrical machines of various structures, while also considering the impact of intricate slot configurations. The Fourier expansion of ${\left[\frac{1}{h_{c}+\delta \left(\theta ,\alpha \right)}\right]}^{2}$ on the interval $\left[-\frac{\pi }{z},\frac{\pi }{z}\right]$ can be expressed as Equation (7):

(7) $${\left[\frac{1}{h_{c}+\delta \left(\theta ,\alpha \right)}\right]}^{2}=G_{0}+\sum_{n=1}^{\infty }G_{k}\cos\left[kz\left(\theta +\alpha \right)\right]$$

where $G_{0}$ and $G_{k}$ are the Fourier coefficients and z is the number of stator slots. Substituting Equations (4) and (7) into Equation (2), the expression of the cogging torque [9] can be simplified as Equation (8):

(8) $$T_{cog}=\frac{\mu_{0}\pi zL_{a}}{4}\left(R_{2}^{2}-R_{1}^{2}\right)\sum_{n=1}^{\infty }kG_{k}F_{n}\sin\left(kz\alpha \right)$$

where $L_{a}$ is the axial length of the armature and $R_{1}$ and $R_{2}$ are the outer radius of the rotor core and the inner radius of the stator separately. *k*, n are positive integers and satisfy $n=\frac{kz}{2p}$, the minimum value of k is $\frac{2p}{GCD\left(2p,z\right)}$.

### 2.2. Heuristic Optimization with SMS-EMOA

**Figure 2:**
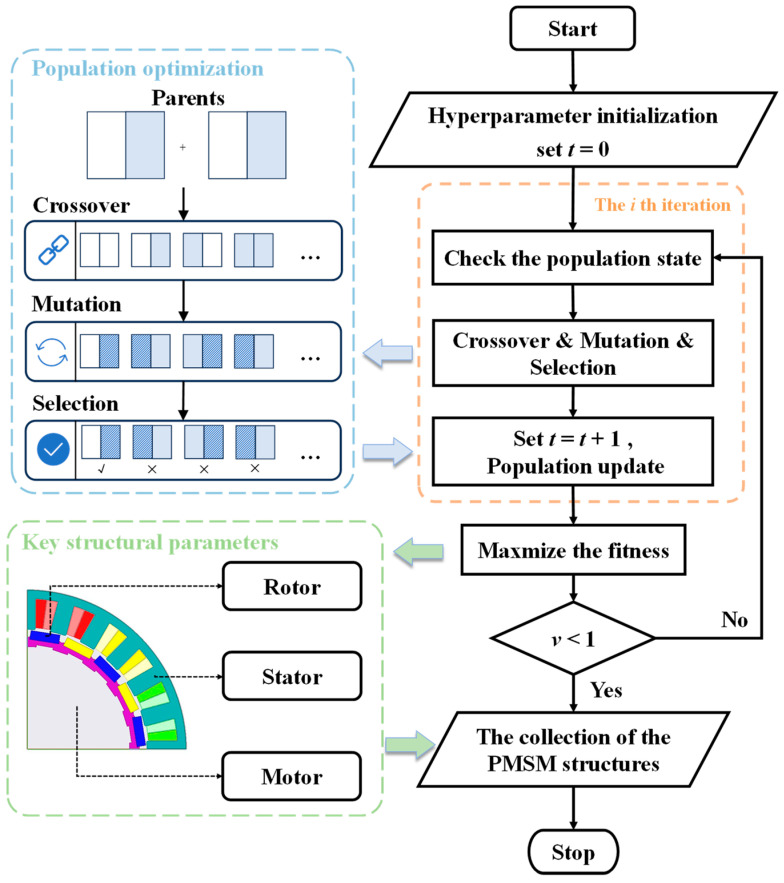
The training step of the SMS-EMOA.

This section provides a brief overview of the fundamental principles of the heuristic optimization algorithm utilized [13,14]. The basic workflow of the SMS-EMOA is depicted in Figure 2. It comprises three core steps: population initialization, genetic mutation, and iteration determination. Through repetitive training, it aims to identify the optimal solution set under the constraints of multiple optimization objectives and boundary conditions [15,16].

#### 2.2.1. Description of the Proposed Optimized Method

The hypervolume measure, also known as the φ metric, is a commonly used quality measure for comparing the outcomes of evolutionary multi-objective optimization algorithms (EMOA). The fundamental Algorithm 1 outlines the process. Beginning with an initial population of μ individuals, a new individual is generated through randomized variation operators. If another individual result in a population of higher quality is replaced according to the *φ* metric, the new individual becomes a member of the subsequent population.
**Algorithm 1.** SMS-EMOA1: *P*_0_ ← init()                                                                                                                         /* Initialize random population of μ individuals */ 2: t ← 0 3: repeat 4:     $q_{t+1}$ ← generate $\left(P_{t}\right)$                                                                                        /* generate offspring by variation */ 5:     $P_{t+1}$ ← Reduce$\left(P_{t}U\{q_{t+1}\}\right)$                                                             /* select μ best individuals */ 6:        t←t+1 7: **until** termination condition fulfilled

The procedure named Reduce, as outlined in Algorithm 2, selects the μ individuals for the subsequent population. NSGA-II [17] is a steady-state algorithm founded on two key principles: non-dominated sorting is utilized as a ranking criterion, and hypervolume is employed as a selection criterion to eliminate individuals that contribute the least hypervolume to the lowest-ranked front. The algorithm fast-nondominated-sort, used in NSGA-II [17], partitions the population into v sets $R_{1},\ldots ,R_{v}$, based on the defined non-dominated sorting. The sets, referred to as fronts, have an index that denotes their hierarchical order based on domination levels, wherein the solutions within each front are mutually non-dominated. The initial subset includes all non-dominated solutions from the original set Q. The second front consists of individuals that are non-dominated in the set (*Q*\*R*_1_), meaning each member of $R_{2}$ is dominated by at least one member of $R_{1}$. In a more general sense, the ith front consists of individuals that remain non-dominated even when individuals from fronts *j* with j<i are removed from Q.
**Algorithm 2.** Reduce(Q)1: $\left[R_{1},\ldots ,R_{v}\right]$ ← fast-nondominated-sort(Q)         /* all v fronts of Q */ 2: r ← ${argmin}_{s\in R_{v}}$ $\left[∆\varphi (s,R_{v})\right]$                                                    /*$s\in R_{v}$ with lowest $∆\varphi (s,R_{v})$ */ 3: **return** (Q\{r})                                                                                                  /* eliminate detected element */

Afterwards, one individual is discarded from the worst ranked front. Whenever this front comprises $\left|R_{v}\right|>1$ individuals, the individual $s\in R_{v}$ is eliminated that can be described as Equation (9):

(9) $$\Delta \varphi \left(s,R_{v}\right)=\varphi \left(R_{v}\right)-\varphi \left(R_{v}\backslash \left\{s\right\}\right)$$

The value of $\Delta \varphi \left(s,R_{v}\right)$ can be interpreted as the exclusive contribution of individual s to the φ metric value of its corresponding front [16]. According to the definition of $\Delta \varphi \left(s,R_{v}\right)$, a dominating individual is always retained, and a non-dominated individual is never replaced by a dominated one. This measure ensures that individuals maximizing the population’s φ metric value are preserved, thereby preventing a decrease in the population’s covered hypervolume through the application of the Reduce operator. Therefore, the following invariant holds for Algorithm 1 that can be described as Equation (10):

(10) $$\varphi \left(P_{t}\right)\leq \varphi \left(P_{t+1}\right)$$

#### 2.2.2. Key Hyperparameter of the Optimizer

(1) Steady-state selection

Due to the significant computational effort involved in calculating the hypervolume, a steady-state selection scheme is employed. Since only one individual is created in each generation, only one individual needs to be removed from the population. Consequently, the selection operator must calculate a maximum of (μ+1)φ metric values (precisely μ+1 values if all solutions are non-dominated). These values correspond to the subsets of the lowest-ranked front, with one point left out in each subset. In contrast, a (μ+λ) selection scheme would necessitate the computation of $\left(\frac{\mu +\lambda }{\mu }\right)$ possible φ metric values to identify an optimally composed population that maximizes the net value of the φ metric. Additionally, the use of a steady-state scheme allows for effortless parallelization through asynchronously distributed function evaluations.

(2) Population size

In contrast to other strategies that archive non-dominated individuals, the SMS-EMOA maintains a population of both non-dominated and dominated individuals at a constant size. Retaining only non-dominated individuals can result in small or single-member populations, leading to a significant loss of population diversity. Modifications in the SMS-EMOA with dynamic population sizes have been explored in [18]. These variants are particularly useful when an acceleration of the initial stages of the evolutionary process is desired. To prevent the loss of diversity, it is recommended that a minimum limit be imposed on the population size.

#### 2.2.3. Handling of Boundary Solutions

In a two-dimensional solution space, the reference point $y_{ref}$ is solely required for calculating the hypervolume between the two extreme points, representing the best and worst objective values, respectively. For simplicity, we have chosen to omit $y_{ref}$ and always retain these extreme solutions. However, when dealing with multiple objective functions, additional points within the boundary may contribute to the hypervolume, depending on the selection of reference points. These boundary points encompass at least one worst objective value. Not all boundary solutions are preserved; their inclusion depends on their respective contributions. To address this, we adopt a dynamic approach to handling the reference points, recalculating $y_{ref}$ for every generation. Specifically, $y_{ref}$ is determined as the vector of the currently worst objective values, increased by 1.0. Consequently, the contribution of a point with a worst objective value is assessed based on the difference to the reference point, with a neutral effect in the product. As a result, favorable non-extreme solutions may exhibit superior hypervolume performance. Initial observations indicate that dynamic reference point handling is preferable to a static approach.

#### 2.2.4. Selection Variants of SMS-EMOA

A comparative analysis of various selection modes within the SMS-EMOA framework was presented in [19]. These approaches all incorporate the concept of hypervolume contribution in conjunction with non-dominated sorting, the shared dominance criterion, or the count of dominating points. In this paper, we will provide more detailed descriptions of the latter approach. The number of dominating points d(s,P(t)) is the number of points from set P(t) that dominate point s, formally can be described as Equation (11):

(11) D(s,P(t))=|{y∈}P(t)|y≺s|

The modified Reduce procedure is illustrated in Algorithm 3. In the case of dominated solutions, the number of dominating points, denoted as d(s,P(t)), is employed as the selection criterion. Conversely, if all individuals in *P*(*t*) are non-dominated, the contributing hypervolume Δφ is utilized. When dealing with a population that consists of multiple fronts, the individual with the highest value of d(s,P(t)) among the solutions in the lowest-ranked front is discarded. In instances where all individuals have a d(s,P(t)) value of zero, the Δφ selection is implemented instead.
**Algorithm 3.** Reduce(Q)1: [$R_{1}$,…,$R_{v}$] ← nondominated-sort(Q)                /* all v fronts of Q */ 2: **if** v>1 **then** 3:        $r\leftarrow {argmax}_{s\in R_{v}}$ [d(s,Q)]                                        /* $s\in R_{v}$ with highest d(s,Q) */ 4: else 5:         r ← ${argmax}_{s\in R_{1}}$ [$∆\varphi (s,R_{1})$]                                /* $s\in R_{1}$ with lowest $∆\varphi (s,R_{1})$ */ 6: end if 7: **return** (Q\{r})                                                                                         /* eliminate detected element */

One motivation for developing such a measure is the smaller runtime complexity compared to that of the hypervolume measure. Additionally, the objective was to achieve a different ranking of dominated solutions, with an emphasis on sparsely filled regions of the solution space. In this approach, individuals are retained in subsequent generations to fill gaps in the approximation of the Pareto front. The contributing hypervolume is utilized to distribute solutions advancing the front on which they reside. Although the ultimate aim is to distribute solutions advancing the first non-dominated front $\left(R_{1}\right)$, this is not the sole objective on other fronts. The measure favors solutions located in regions where the superior fronts are sparsely populated. The idea behind this is that offspring solutions can progress to higher-ranking fronts and occupy those vacancies. In densely populated regions of the non-dominated front, it is not beneficial to retain dominated individuals.

#### 2.2.5. Calculation of Contributing Hypervolume ∆φ

The best-known algorithms compute the hypervolume with a runtime that is polynomial in the number of points but exponentially increases with the number of objectives. Moreover, dedicated algorithms have been developed to efficiently calculate all values of Δφ in cases of two and three objectives simultaneously. These specialized algorithms are substantially faster than repeatedly calling procedures to compute the overall hypervolume.

In the case of two objectives, the points from the front with the lowest rank among the non-dominated ones are examined, and they are arranged in ascending order according to the values of the first objective function, $f_{1}$. As a result, a sequence is obtained that is sorted in descending order based on the $f_{2}$ values, since the points are mutually non-dominated. To calculate Δφ for a sorted front $R_{v}=\{s_{1},\ldots ,s_{\left|R_{v}\right|}\}$, the following formula is used $\left(i=2,\ldots ,\left|R_{v}\right|-1\right)$:

(12) $$\Delta \varphi \left(s_{i},R_{v}\right)=\left(f_{1}\left(s_{i+1}\right)-f_{1}\left(s_{i}\right)\right)\cdot \left(f_{2}\left(s_{i-1}\right)-f_{2}\left(s_{i}\right)\right)$$

The paper [18] presents a rapid algorithm for computing all contributions in a three-objective space. This algorithm has a runtime of $O\left(\mu^{3}\right)$ and relies on a two-dimensional projection of the point set. Specifically, the $f_{1}-f_{2}$ plane is divided into a grid based on the coordinates of all μ+1 solution vectors, including the reference point. For each grid cell $c_{ij}\left(i=1,\ldots ,\mu +1,j=1,\ldots ,\mu +1\right)$, the μ+1 values of $f_{3}$ are computed at the corner point dominating it in the $f_{1}-f_{2}$ plane. Based on these values, the best value, $h_{1}\left(c_{ij}\right)$, and the second-best value, $h_{2}\left(c_{ij}\right)$, are determined. Storing this information in two matrices of dimensions (μ+1)×(μ+1), the exclusive contribution of the ith solution vector (i=1,…,μ+1) can be calculated as follows. For any grid cell $c_{ij}$ with the $h_{1}\left(c_{ij}\right)$ value determined by the ith solution, the volume $v_{ij}$ is obtained by multiplying the surface area of $c_{ij}$ with the difference $\left|h_{1}\left(c_{ij}\right)-h_{2}\left(c_{ij}\right)\right|$. By accumulating the values of $v_{ij}$, the exclusive contribution of the *i*th solution to the dominated hypervolume can be determined. The $O\left(\mu^{3}\right)$ runtime arises due to the quadratic number of cells and the linear-time calculations for each cell value. While it may be possible to further optimize this algorithm, such as through incremental updates, the performance of the algorithm in the computations performed in this paper is reasonably fast. Another potential advantage is the non-recursive nature of the algorithm and its use of simple data structures, making it relatively easy to implement and debug.

### 2.3. PMSM Structure Optimization Based on SMS-EMOA

**Figure 3:**
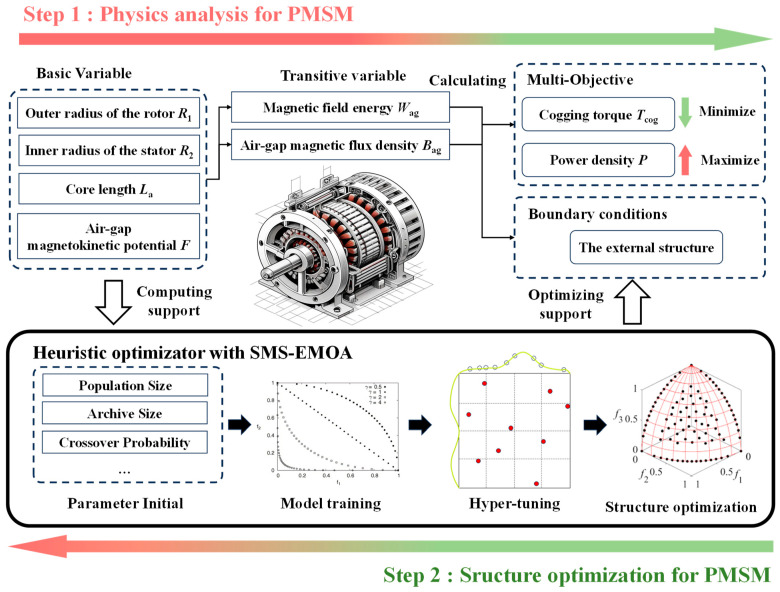
Flowchart of PMSM-SLA modelling. Reprinted with permission from Ref. [10], 2007, @ European Journal of Operational Research.

Based on the analysis of motor structural physical factors conducted in Section 2.1, the decision variables in the optimization design should include the inner and outer diameters of the motor’s stator and rotor, the number of pole pairs, winding turns, the pole arc coefficient, and the skew angle. The optimization objectives are to reduce cogging torque and increase the motor’s power density. The two-step modelling flowchart is shown in Figure 3.

## 3. Numerical Example

### 3.1. Experimental Setup

To verify the effectiveness of the proposed method, this paper conducts case studies on a surface-mounted permanent magnet synchronous motor (SPMSM) of the 20p24s model. Initially, a simulation model of the motor is established based on a finite element model, and the motor efficiency under various structural parameters is calculated.

#### Motor Simulation Calculation

**Figure 4:**
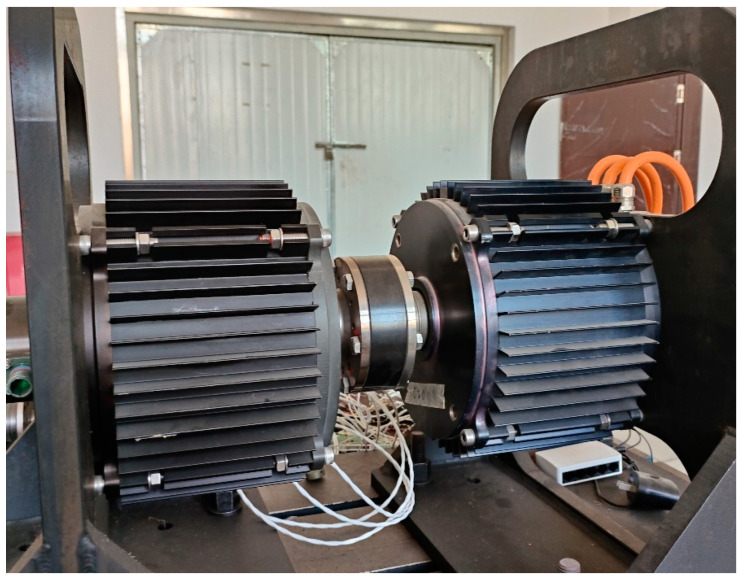
The prototype test platform of the 20p24s SPMSM.

The prototype of the 20p24s SPMSM is shown in Figure 4. The main parameters are summarized in Table 1.

The simulation experiments for this motor were conducted. The magnetic field lines are reasonable, and the magnetic density distribution is correct.

### 3.2. SPMSM Structure Optimization with SMS-EMOA

#### 3.2.1. Procedure of the Multi-Objective Optimization with SMS-EMOA

In this section, the SMS-EMOA is applied to search the optimal combination of pole–arc coefficients of magnetic poles [20]. The key steps are summarized as Algorithm 4.
**Algorithm 4.** Fast Non-dominated Sort1:    Initialize the sets and counters for each individual p∈P 2:    **for** each individual p∈P **do** 3:        p.sp ← {} {Initialize the set of dominated solutions for p} 4:        p.np ← 0 {Initialize the domination counter for p} 5:    end for 6:    F1←{} {Initialize the first Pareto front} 7:    **for** each individual p∈P **do** 8:            **for** each individual q∈P **do** 9:                 **if** p dominates q **then** 10:                     Add q to p’s set of dominated solutions: p.sp←p.sp∪{q} 11:             **else if** q dominates p **then** 12:                            Increment domination counter for p:p.np←p.np+1 13:             **end if** 14:        **end for** 15:        **if** p.np==0 **then** 16:                Assign rank 1 to p:p.rank←1 17:                Initialize crowding distance for p:p.crowding distance←0 18:                Add p to the first Pareto front: F1←F1∪{p} 19:        **end if** 20:    end for 21:    F←{F1} {Initialize the set of Pareto fronts} 22:    i←1 {Initialize Pareto front counter} 23:    **while** |F[i−1]|>0 **do** 24:         Q←{}{Initialize the next Pareto front} 25:                **for** each individual p∈F[i−1] **do** 26:                    **for** each individual q∈p.sp **do** 27:                         Update domination counter for q:q.np←q.np−1 28:                         **if** q.np==0 **then** 29:                                 Assign rank i+1 to q:q.rank←i+1 30:                                 Initialize crowding distance for q:q.crowding_distance←0 31:                                 Add q to the next Pareto front: Q←Q∪{q} 32:                         **end if** 33:                    **end for** 34:                **end for** 35:            i←i+1{Increment Pareto front counter} 36:                Add the next Pareto front to the set of Pareto fronts: F←F∪{Q} 37:    end while 38:    **return** Pareto fronts F with $T_{cog}$ given by:                                                                 $T_{cog}=\frac{\mu_{0}\pi zL_{a}}{4}\left(R_{2}^{2}-R_{1}^{2}\right)\sum_{n=1}^{\infty }kG_{k}F_{n}\sin kz\alpha$


In the context of evolutionary algorithms, the optimization process begins with sampling, which establishes an initial solution set, utilizing random sampling methods and initializing a Population object with either new or pre-evaluated variables. Selection mechanisms then determine mating pairs for producing offspring, employing strategies such as random selection, neighborhood-based selection, or tournament selection to introduce selection pressure. Crossover operations generate offspring by combining selected parents, while mutation operations, typically initialized with a specific probability, are applied to increase population diversity and enhance performance. The algorithm includes a feature to eliminate duplicate solutions post-crossover and mutation, ensuring a unique set of offspring. The offspring parameter controls the number of offspring generated, with the default setting producing offspring equal to the population size, although it can be adjusted to achieve a steady-state algorithm version.

#### 3.2.2. Definition of the Optimization Space

After defining the basic process of multi-objective optimization, it is necessary to further clarify the optimization objectives and boundary conditions. The objective function and associated constraints can be determined with Equation (13):

(13) $$\min T\left(\alpha_{p}\right)=\min\left\{\max T_{cog}\left(\alpha \right)\right\}\;0.835\leq \alpha_{p}\leq 0.865$$

It can be seen that the determined tilt angle basically satisfies $\sin\frac{bkz\gamma }{2}=0$. Consequently, the optimal pole–arc coefficients of the adjacent magnetic poles can be determined using Equation (14):

(14) $$\left\{ \begin{array}{l} \alpha_{p1}+\alpha_{p2}=2\alpha_{p}\\ \cos\frac{n\pi }{4}\left(\alpha_{p1}-\alpha_{p2}\right)=0 \end{array}\right.$$

#### 3.2.3. Approaching for Pareto Optimal Solution

**Figure 5:**
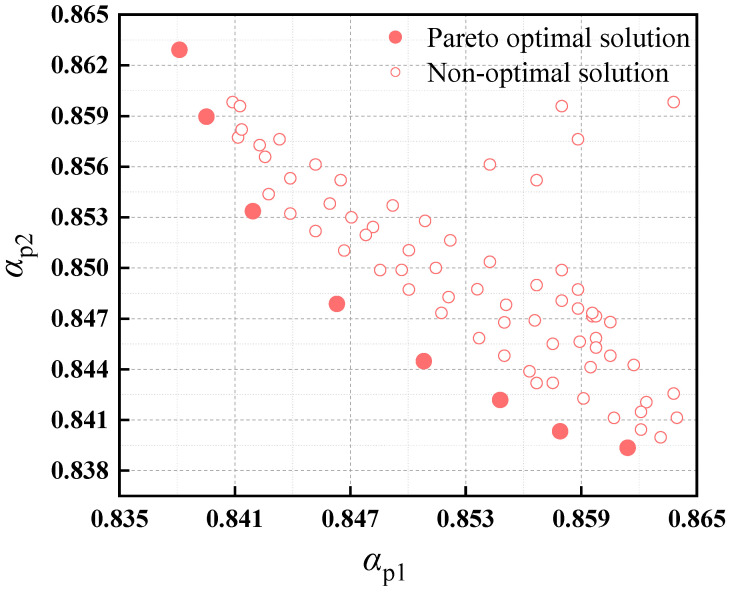
Objective value distributions and Pareto-front points.

The SMS-EMOA produces a Pareto front that represents all optimal solutions from the last generation [21]. The number of dominating points can be efficiently calculated by comparing the selection candidates with all solutions to check for dominance. This process has a time complexity of $O\left(m\mu^{2}\right)$, where m represents the number of objectives and μ is the population size. The expected runtime complexity is lower than that of the basic SMS-EMOA algorithm. While the worst-case complexity remains the same, as the population may exclusively consist of non-dominated solutions, the average-case complexity is assumed to be better. Figure 5 displays the optimization results for PMSM, with a distinct Pareto front evident at the bottom of the graph. An optimal solution is selected and substituted into the iterative formula for verification.

Multiple results on standard benchmark problems demonstrate that the SMS-EMOA is well-suited for Pareto optimization with two and three objectives. It consistently outperforms established techniques such as SPEA2 [22], *ϵ*-MOEA, and NSGA-II [17] in terms of both convergence and φ metric values. The examples reveal a well-distributed set of results on the Pareto front, with particular emphasis on the boundaries and regions around knee points. A notable feature of the SMS-EMOA is its ability to approximate the Pareto set with a small number of individuals, which is frequently desirable in practice.

A novel selection criterion has been developed to supplant hypervolume-based selection in the presence of dominated solutions within the population. This criterion evaluates a solution by considering the number of solutions that dominate it, directing the evolutionary search toward less explored regions near the Pareto front. Both variants of the SMS-EMOA (the basic algorithm and the coupling with the number of dominated points criterion) outperformed conventional strategies, implying the effectiveness of the hypervolume selection mechanism irrespective of the specific details of the selection scheme.

The SMS-EMOA not only improved the distributions of solutions but also identified solutions that clearly dominate the baseline design. Furthermore, the SMS-EMOA was coupled with a metamodeling tool, which provided fast fitness function approximations to save costly exact evaluations in unfavorable regions. The SMS-EMOA was the first algorithm to consistently produce solutions that dominate the baseline design. Moreover, this algorithm produced robust solutions.

In these cases, the runtime of the operations performed within the EMOA can almost be neglected, and the SMS-EMOA is certainly a well-suited optimizer.

### 3.3. Results and Discussion

#### 3.3.1. Optimization Results and Verification

**Figure 6:**
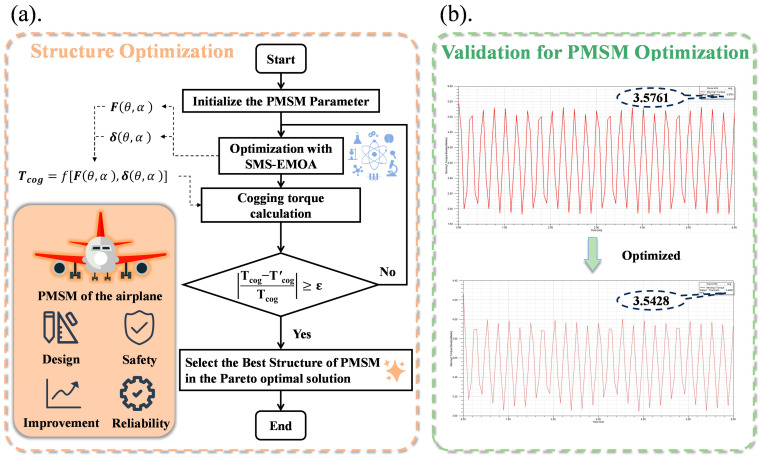
Flowchart of cogging torque calculation. (**a**) structure optimization. (**b**) validation for PMSM optimization.

Figure 6 shows the flowchart for calculating cogging torque. The cogging torque can be calculated using the SPMSM motor’s data. A data type that reduces the cogging torque is needed. Therefore, the flowchart demonstrates that the data require a loop condition where the loop will be exited, and the desired optimal solution will be outputted when the optimized data meet the condition.

Firstly, the relevant data of the motor, including winding, magnetic field, and geometric features, need to be collected. Then, based on this data, an initial calculation of the cogging torque will be performed.

Next, a loop is entered that includes the optimization of motor parameters. By fine-tuning the parameters in each iteration of the loop, the cogging torque can be gradually reduced.

In each iteration of the loop, the cogging torque needs to be calculated by using the optimized parameters. If the calculated cogging torque satisfies the predefined reduction condition, the loop will be exited, and the optimal solution will be outputted.

If the cogging torque does not meet the reduction condition, a further adjustment of the parameters and recalculation are required until the condition is fulfilled [23].

In summary, this flowchart describes the process of calculating cogging torque and presents a loop condition to optimize and output the optimal solution. Through this process, data can be obtained that effectively reduce cogging torque to improve the motor’s performance.

As shown in Figure 6a, $T_{cog}$ represents the initial cogging torque, and $T_{cog1}$ represents the cogging torque obtained after optimization. Setting the value $\varepsilon =1\times 10^{-3}$, as verified by Figure 6b, it can be confirmed that

(15) $$\left|\frac{T_{cog}-T_{cog1}}{T_{cog}}\right|=0.00931182>\varepsilon$$

The optimum value of PMSM is selected, and the loop is exited after the values have been outputted.

Through the data set calculation analysis, the final motor optimization results are shown in Table 2.

#### 3.3.2. Hyperparameter Optimization

**Figure 7:**
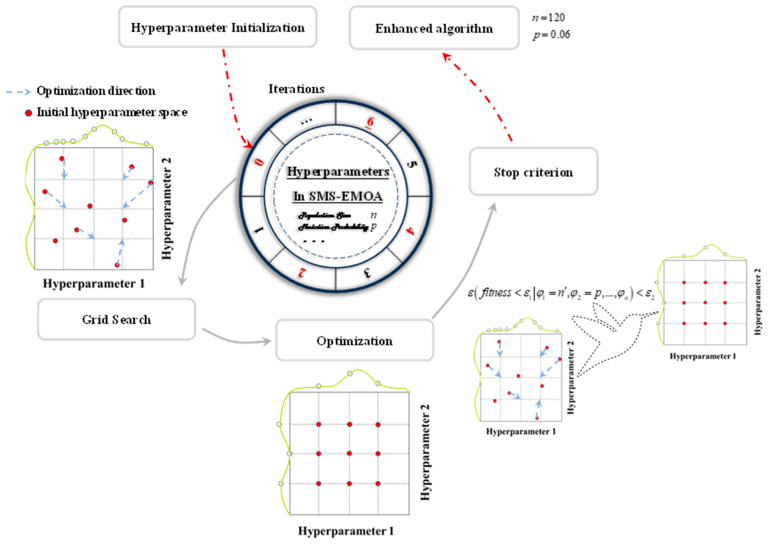
Hyperparameter optimization.

Model parameters define how the input data are transformed into the desired output and are learned during training to optimize a loss function [24]. On the other hand, hyperparameters, which influence the structure of the model, cannot be directly trained from the data [25]. Instead, they are used to tune the model and improve its performance. Various methods exist for adjusting the hyperparameter space to achieve an optimal model when using a single algorithm [26,27]. Grid search and random search are commonly used approaches for exploring the hyperparameter space and finding the best model configuration. Grid search involves building a model for each possible combination of hyperparameters in a pre-defined grid and selecting the model with the best performance. While grid search can be exhaustive, it may also be inefficient. In contrast, random search randomly samples hyperparameter values from a statistical distribution, rendering it less restrictive. Random search assumes that all hyperparameters are not equally important and tends to work well in practice. Another approach to tune hyperparameters is through a genetic algorithm, as introduced in this work. Figure 7 illustrates the steps involved in the genetic algorithm-based hyperparameter tuning method. Initially, an initial population of models is generated by randomly selecting hyperparameters. The models in the population are evaluated based on a loss function that measures the discrepancy between the model’s predicted values and the true values. A new population is created using the following steps:

(I) The best models from the previous generation, those with the lowest error, are selected. These models have performed well and are taken as the foundation for creating the next generation.
(II) With a certain probability, crossover is performed to create offspring. During crossover, the hyperparameters of two parent models are combined to produce a new model. If crossover is not performed, the offspring will be an exact copy of its parent.
(III) The latest offspring undergo mutation, with a certain probability. During mutation, the hyperparameters of the model are slightly changed. This introduces diversity in the population, allowing for an exploration of different regions of the search space.
(IV) The offspring are added to the new population, joining the models from the previous generation.

The newly generated population is then used for further optimization. The process of selection, crossover, and mutation is repeated for several generations.

Furthermore, a certain elite population is retained based on an elite percentage. These elite models represent the top-performing individuals and are given priority in the next generation. This helps to maintain the best solutions and prevent regression.

The process continues until the specified number of generations is reached. At the end, the hyperparameters of the best model obtained throughout the generations are reported as the optimal configuration.

Overall, this algorithm combines elements of evolutionary computation, such as selection, crossover, and mutation, to iteratively search for the best set of hyperparameters for a given problem.

The model tuning process is depicted in the flowchart shown in Figure 7. Initially, the raw dataset is obtained, and subsequently, feature engineering is performed to extract features and targets employing domain knowledge. Following this, the processed dataset is partitioned into training, test, and validation datasets. The model is initially constructed using either default base learners or previously tuned base learners trained on the training datasets. The performance of the trained model is assessed using the test dataset. In the event that the further optimization of the base learners is required based on prediction accuracy, additional hyperparameter optimization is carried out. If no further optimization is deemed necessary, the model is validated using a separate validation dataset that was not utilized during the model training. If the results meet the desired criteria, the model is saved for future use. Otherwise, the hyperparameter optimization process is repeated. This iteration continues until an acceptable model is obtained. Figure 7 demonstrates the use of grid search and random search for hyperparameter tuning. Although these techniques are effective, they have some disadvantages compared to manual tuning.

Due to its attempt to try every combination of hyperparameters and select the best combination based on cross-validation scores, grid search becomes extremely slow [28,29,30].

The limitation of random search is its inability to ensure the identification of the optimal parameter combination. Consequently, in the conventional tuning process, algorithms are trained through the manual inspection of randomly generated hyperparameter sets, and ultimately, the parameter configuration that aligns most effectively with our objectives is selected.

In this section, the population size is set to 120 and the mutation probability to 0.06, and the innovativeness of SMS-EMOA is confirmed through validation.

## 4. Conclusions

The optimization of motor structure parameters can significantly weaken cogging torque. A segmented skewed magnet pole design was developed to weaken cogging torque, and the optimal solution for different combinations of pole–arc coefficients was summarized using the SMS-EMOA. Finally, the cogging torque of the 20p24s slot–pole combination in a permanent magnet synchronous motor was analyzed, and the results showed that adopting segmented magnetic poles with different combinations of pole–arc coefficients can significantly weaken the cogging torque while having minimal impact on other motor performances. 

The paper focuses on the hyperparameter optimization section, which demonstrates that manual tuning is crucial in determining the most suitable parameters. In order to further advance research, it is recommended that additional algorithmic variants be explored and a comprehensive analysis of strategy parameters conducted.

However, the SMS-EMOA may not be suitable for higher dimensional problems, which typically require a large number of function evaluations. Nonetheless, there are many real-world applications that only allow for a limited number of evaluations due to the expensive simulations that govern the optimization runtime of the PMSM. In such cases, the computational time taken by the operations carried out within the EMOA can be considered negligible, and the SMS-EMOA is undoubtedly an effective optimizer.

## Figures and Tables

**Table 1:** The main structure 31kW of the 20p24s SPMSM.

Parameters	Unit	Range of the Value
Rated power	**kW**	**28~35**
Length of armature	**mm**	**75~85**
Rated speed	**r/min**	**1800~2100**
Number of poles	**-**	**15~25**
Number of slots	-	**20~30**
Magnet thickness	**mm**	**4.9~5.8**
Pole-arc coefficient	-	**0.835~0.865**

**Table 2:** Final motor optimization results.

Parameters	Unit	Range of the Value
Rated power	**kW**	**31**
Length of armature	**mm**	**80**
Rated speed	**r/min**	**2000**
Number of poles	-	**20**
Number of slots	-	**24**
Magnet thickness	**mm**	**5.6**
Pole-arc coefficient		**0.855**

## Data Availability

The data presented in this study are available on request from the corresponding author.
